# Prestige and Dominance: How eWOM Differs Between Consumers High in Authentic and Hubristic Pride

**DOI:** 10.3389/fpsyg.2022.898946

**Published:** 2022-06-15

**Authors:** Jiayao Liu, Qingyun Xiong, Jianan Zhong

**Affiliations:** Department of Psychology and Behavioral Sciences, Zhejiang University, Hangzhou, China

**Keywords:** electronic word-of-mouth (eWOM), authentic pride, hubristic pride, prestige, dominance

## Abstract

Electronic word-of-mouth (eWOM) influences consumers' purchase decisions, but few studies have investigated the antecedents that lead consumers to create different types of eWOM. From the perspective of social interactions, this research explored how two subtypes of pride not only compel consumers to create eWOM but also differently impact four types of eWOM and their mechanisms. Study 1 manipulated the pride state and found that authentic pride promoted positive eWOM and constructive eWOM, while hubristic pride promoted negative eWOM and destructive eWOM. Study 2 examined the effect of pride on eWOM at the trait level and tested the mediating effect of their use of social status pursuit strategy. Overall, this study increases the understanding of different types of eWOM and broadens the literature of the effect of pride and social status pursuit strategy in the context of consumption.

## Introduction

Electronic word-of-mouth (eWOM) refers to statements about a product or company that are made by potential, actual or former customers and available via the internet to many individuals and institutions (Hennig-Thurau et al., 2004; Antonetti et al., 2020), and it is well-established that eWOM often influences consumer purchasing choices. Given its importance, researchers have studied various aspects of eWOM, including factors that drive consumers' eWOM behaviors (Taylor et al., 2012; Hayes and King, 2014; Anggraeni and Diandra, 2017; Nikolinakou and King, 2018b; Chu et al., 2019; Zhou et al., 2020), eWOM's impact on consumers (Godes and Mayzlin, 2004; Chevalier and Mayzlin, 2006; Moe and Trusov, 2011), and how businesses manage and respond to eWOM (Li, 2018; Gössling et al., 2019). However, research on drivers has focused on both general eWOM and positive eWOM, but few studies have explored the factors that lead to different types of eWOM (Hu and Kim, 2018; Nam et al., 2020; Sohaib et al., 2020; Kim and Hwang, 2022). Because different types of eWOM have different impacts on consumers (Lis and Fischer, 2020), clarifying the factors that influence specific types of eWOM will help better manage the business. Previous studies have explored when consumers are more likely to engage in positive eWOM behavior and when they are more likely to engage in negative eWOM behavior, in terms of motivation (Hu and Kim, 2018; Nam et al., 2020), individual differences (Sohaib et al., 2020) and product attributes (Kim and Hwang, 2022). However, there has been no research on what type of eWOM consumers are more likely to post from an emotional perspective. eWOM can be regarded as a specific type of social interaction, and different types of eWOM can help consumers achieve different goals, such as revenge, comfort search, and social engagement-seeking (Wetzer et al., 2007; Dolan et al., 2019). These different eWOM behaviors will leave different impressions on third-party observers (e.g., other consumers), which in turn will have an impact on their influence and social status (Yin and Smith, 2021). Therefore, considering that pride is the emotion most relevant to the acquisition of social status (Cheng et al., 2010; Sznycer et al., 2017), this research explores the influence of pride on different types of eWOM behaviors.

eWOM can be divided into different types. Based on its valence, eWOM can be divided into positive eWOM or negative eWOM (Berger, 2014; Nam et al., 2020). Based on its purpose, eWOM can be divided into venting, revenge taking, entertaining, helping companies, solution seeking, support seeking, and social engagement seeking (Dolan et al., 2019; Weitzl, 2019). These divisions can be further classified into constructive eWOM or destructive eWOM (Wetzer et al., 2007). Given the different impacts of different types of eWOM on consumers and merchants, we believe that it is very important to identify which consumers are more likely to post the types of eWOM that negatively impact merchants.

Several studies have shown that emotions greatly influence individual behaviors (Griskevicius et al., 2010; Campos et al., 2013; Shiota et al., 2014; Nikolinakou and King, 2018a) and eWOM (Berger and Milkman, 2012; Nikolinakou and King, 2018a). Compared with general positive and negative emotions, discrete emotions may better explain why consumers post different types of eWOM, because each discrete emotion may correspond to a unique adaptation mechanism to solve a specific problem faced by early humans (Xu et al., 2021), and overgeneralizing emotions would mislead both researchers and practitioners (Griskevicius et al., 2010). Thus, we focus on the discrete positive emotion of pride, which is important in individual pursuits of social status (Cheng et al., 2010) and drives individuals to send signals that help increase their status (e.g., consumers can become opinion leaders by publishing eWOM; Dalman et al., 2020).

Based on the dominance-prestige model of status (Henrich and Gil-White, 2001), we attempt to explore the impact of different types of pride (authentic pride vs. hubristic pride) on their intentions to create different types of eWOM and the mediating role of social status pursuit strategies. The dominance–prestige model suggests that the pursuit of social status includes prestige strategies, which entails obtaining an increased social status by being recognized and respected due to personal skills or knowledge (Henrich and Gil-White, 2001), and dominance strategies, which entails obtaining an increased social status by possessing resources and controlling profits (Anderson and Kilduff, 2009). Previous research has demonstrated that authentic pride leads to prestige strategies and hubristic pride leads to dominance strategies (Cheng et al., 2010; Tracy et al., 2020). Furthermore, the use of different social status pursuit strategies leads to different behaviors (Conlon, 2019; Panchal and Gill, 2020; Ketterman and Maner, 2021; King and Auschaitrakul, 2021). In summary, we believe that consumers with a high level of authentic pride should be more inclined to adopt prestige strategies, and then engage in positive eWOM and constructive eWOM, because these are value-cocreation behaviors that can also bring benefits to businesses. On the contrary, consumers with a high level of hubristic pride are more inclined to adopt dominance strategies, and then carry out negative eWOM and destructive eWOM, because these are value-codestruction behaviors that will harm the merchants.

In this study, we make several theoretical contributions to the literature. First, our work identifies the types of eWOM created by consumers with different emotions, thereby improving the extant understanding of eWOM. Previous studies have focused on the factors that promote overall eWOM but have not paid attention to the specific types of eWOM that consumers post (Nam et al., 2020; Miranda and Duarte, 2022). Second, we enrich the existing research on emotions in eWOM contexts, specifically, on discrete emotions, pride. Our results show that different subtypes of pride can determine different eWOM behaviors. Finally, we regard eWOM as an act of building and maintaining social status in shopping-related fields and find that use of social status pursuit strategy influences which types of eWOM consumers want to write, thereby expanding the knowledge of eWOM behaviors.

## Literature Review and Theoretical Development

### Different Types of eWOM

Word-of-mouth (WOM) refers to informal communications between consumers about products or services (Anderson, 1998). Due to the rapid development of the internet, consumers can share their opinions on products and services with many other consumers through social network platforms and websites, that is, through eWOM (Hennig-Thurau et al., 2004). The unprecedented speed of eWOM propagation allows multidirectional information exchanges between communicators and receivers (Cheung and Thadani, 2012). It is therefore easier to access eWOM than traditional WOM, and the former method is also more effective. Notably, there are multiple types of eWOM, and different types of eWOM have different effects (Weitzl, 2019).

According to its valence, eWOM can be divided into positive or negative eWOM. Generally, the valence of eWOM shared by consumers is consistent with their experience (Berger, 2014). A satisfactory consumption experience leads to positive eWOM, and an unsatisfactory consumption experience leads to negative eWOM (Nam et al., 2020). However, there are also factors that make some consumers more likely to create positive eWOM (Miranda and Duarte, 2022) and others more likely to write negative eWOM after a similar consumption experience. For example, Zhang et al. (2014) have found that women have strong relationships with others are more likely to spread negative eWOM, even at risk of damaging their self-image. Their research has also found that interdependent self-construction consumers are more likely to spread negative WOM at risk of being deemed unwise. In addition, consumers with a maximization strategy may share positive reviews of an unsatisfactory consumption experience with close others to obtain better results through comparisons (Olson and Ahluwalia, 2021).

According to the purpose for its creation, eWOM can be divided into constructive or destructive eWOM. Wetzer et al. (2007) first proposed the constructive-destructive dimension based on the belief that there is a specific link between the emotions that are aroused by a product or service failure and consumers' reasons for writing eWOM (constructive eWOM vs. destructive eWOM). For example, angry consumers try to vent or take revenge, while disappointed and regretful consumers hope to warn others or strengthen social ties. When consumers experience a service failure, they may communicate with companies in different ways on social media. Some consumers will use a “good response,” such as directly contacting a company and then sharing how well the company resolved their complaint(s). In contrast, some consumers will use a “bad response,” such as spreading negative eWOM without contacting a company, complaining to others, or sharing how the company was unable to resolve their complaint(s) (Grégoire et al., 2015). According to their purpose, such responses can thus be identified as constructive eWOM when consumers seek solutions to problems or to rebuild relationships between customers and brands, or as destructive eWOM when consumers tell others to avoid certain brands' goods or services, thereby harming these companies (Weitzl, 2019). That is, constructive eWOM is an act of value-cocreation, while destructive eWOM is an act of value-codestruction (Dolan et al., 2019).

Previous research has investigated several factors that promote eWOM from the perspective of consumer interaction, such as affiliation, altruism and self-enhancement (Schutz, 1958). Social connections (strengthening existing relationships and creating new relationships) are also an important motivation for people to convey eWOM through social media (Hayes and King, 2014). Consumers will recommend brands and write online reviews to guide others' purchase decisions (Lovett et al., 2013) by sharing content that expresses their concerns about, appreciation for, or encouragement to purchase a good or service from others (Phelps et al., 2004). eWOM also articulates and strengthens a consumer's personality when he or she shares advertisements that are consistent with his or her personality (Taylor et al., 2012). However, few studies have demonstrated what factors compel consumers to create different types of eWOM (Hu and Kim, 2018; Nam et al., 2020; Sohaib et al., 2020; Kim and Hwang, 2022). Hu and Kim (2018) have found that there are different motivations behind positive eWOM and negative eWOM. Nam et al. (2020) have found that whether expectations are confirmed determines whether consumers will write positive eWOM or negative eWOM. Sohaib et al. (2020) have found that promotion-focused customers are more likely to spread positive eWOM, while prevention-focused customers are more likely to spread negative eWOM. Kim and Hwang (2022) have found that authenticity is influential for positive eWOM only, and value is influential for negative eWOM only. However, no research has been conducted to explore the emotional factors that lead to different types of eWOM.

### Pride and the Different Types of eWOM

Many studies have shed light on the impacts of emotion on cognition and behavior in the field of consumption. For example, positive emotions can promote individuals' variety seeking (Kahn and Isen, 1993) and impulsive buying behaviors (Weinberg and Gottwald, 1982). Anxiety can cause individuals to delay their decision-making (Hafner et al., 2016). Awe can compel consumers to share video advertisements (Nikolinakou and King, 2018a), while anger will promote consumers' retaliation (Antonetti et al., 2020). In the present work, we extend this literature by examining how pride can influence eWOM. Because eWOM can be regarded as a way to obtain social status in the shopping field (Dalman et al., 2020), and pride is the emotion most relevant to obtaining social status (Cheng et al., 2010; Sznycer et al., 2017).

Pride is a kind of positive, self-conscious emotion. One feels pride when he or she achieves success and attributes it to himself or herself (Weiner, 1985). Pride can moderate individuals' behaviors and help them achieve long-term goals. It can help individuals establish lasting personal resources and attain greater achievements (Fredrickson, 2001). From an evolutionary perspective, pride is an adaptive psychological mechanism that is produced when individuals compete with other group members for social status (Cheng et al., 2013; Sznycer et al., 2017). Pride motivates people to work harder to obtain and maintain social status. For example, activating individuals' pride will increase their desire to receive attention from others (Griskevicius et al., 2010), and individuals who experience pride are more likely to successfully perform tasks (Williams and DeSteno, 2008).

Pride also prompts individuals to send signals of their high social status through a set of spontaneous non-verbal expressions, including a small smile, a slight backward tilt of the head, an expanded posture, or having one's arms akimbo with the hands on the hips (Tracy and Robins, 2004, 2008). These clues can imply an improvement in social status, and even blind athletes have these tendencies (Tracy and Matsumoto, 2008). An additional study has also shown that the performance of pride is linked to high status (Shariff et al., 2012). Accordingly, we believe that pride drives consumers to engage in eWOM, a specific form of social interaction, to pursue increased social status.

Previous research has shown that pride has a positive impact on eWOM creation (Wen et al., 2018), but the current research aims to test whether different subtypes of pride will drive consumers to create different types of eWOM. Based on previous studies, Tracy and Robins (2007) distinguished prosocial, achievement-oriented pride from the self-aggrandizing, hubristic form of this emotion to develop its two subtypes: authentic pride and hubristic pride. When individuals attribute success to unstable and controllable factors, they will show greater authentic pride; when individuals attribute success to stable and uncontrollable factors, they will show greater hubristic pride. Previous research has shown that authentic pride and hubristic pride have a unique relationship with various personality variables and status variables (Dickens and Robins, 2020). In terms of personality, authentic pride is positively correlated with agreeableness, conscientiousness, extraversion, openness, self-esteem, proactive coping and self-efficacy, whereas it is negatively correlated with neuroticism, anxiety and loneliness, which indicates a healthy function. However, hubristic pride is negatively correlated with agreeableness, conscientiousness and self-esteem, whereas it is positively correlated with depression and loneliness. In terms of the acquisition of social status, both authentic pride and hubristic pride are associated with higher perceived social status, but with different social status pursuit strategies: authentic pride is more strongly associated with prestige, while hubristic pride is more strongly associated with dominance (Cheng et al., 2010).

Based on the relevant literature on pride, we believe that different types of pride can lead to different types of eWOM behaviors. First, authentic pride and hubristic pride are differently related to personality variables, and consumers with distinct personality patterns may create different types of eWOM. For example, authentic pride has a positive correlation with agreeableness, which makes people more likely to forgive (McCullough and Hoyt, 2002; Neto, 2007), mitigates their negative feelings, judgments, and behaviors toward a business (Enright et al., 1992), reduces their motivation to take revenge and avoid the business, and enhances their motivation to be kind to offenders (McCullough, 2000). Therefore, we believe that authentic pride leads to consumers being more likely to post positive eWOM and constructive eWOM as a friendly signal. However, individuals who are higher in hubristic pride are more impulsive (Carver et al., 2010), which is highly correlated with aggressive behavior (García-Forero et al., 2009). Therefore, we believe that consumers with high hubristic pride are more likely to write negative eWOM and destructive eWOM as an act of aggression.

Second and more importantly, authentic pride and hubristic pride can lead individuals to adopt different strategies for acquiring social status, which in turn lead them to engage in different types of eWOM behaviors (we will elaborate on this as a mediation mechanism in the next section). Briefly, individuals high in authentic pride are more likely to adopt a prestige strategy (Cheng et al., 2010), which leads consumers to demonstrate their friendliness and potential benefits by posting positive eWOM and constructive eWOM, while individuals high in hubristic pride are more likely to adopt a dominance strategy (Cheng et al., 2010), which leads consumers to intimidate merchants and others who see the reviews by posting negative eWOM and destructive eWOM.

Accordingly, we believe that authentic pride will promote positive and constructive eWOM behaviors and that hubristic pride will promote negative and destructive eWOM behaviors. Hence, we posit the following:

H1a: Authentic pride positively predicts the intention to write positive eWOM.
H1b: Hubristic pride positively predicts the intention to write negative eWOM.
H2a: Authentic pride positively predicts the intention to write constructive eWOM.
H2b: Hubristic pride positively predicts the intention to write destructive eWOM.

### The Mediating Effect of Social Status Pursuit Strategies

Considering that eWOM behavior can be regarded as an act of gaining and maintaining social status in the shopping world, we propose the mediating effect of the use of social status pursuit strategies based on the dominance-prestige model of status (Henrich and Gil-White, 2001). The dominance–prestige model suggests that the pursuit of social status includes dominance strategies, which are common in primates, and prestige strategies, which are unique to humans. Dominance entails obtaining an increased social status by possessing resources and controlling profits (Anderson and Kilduff, 2009). Such status is acquired and maintained through the use of power, fear, intimidation and coercion (de Waal-Andrews et al., 2015). Prestige, on the other hand, entails obtaining an increased social status by being recognized and respected due to personal skills or knowledge (Henrich and Gil-White, 2001). In general, dominance is a strategy of pursuing social status by increasing costs for others, while prestige is a strategy of pursuing social status by providing benefits to others (Henrich and Gil-White, 2001; von Rueden et al., 2011).

Previous research has suggested that many factors influence which social status pursuit strategies people use, such as physical size, skills and the situation (Tracy et al., 2020). However, pride, as an automatic affect program that allows individuals to cope most effectively with opportunities for rank attainment, directly guides individuals' use of status strategies. Authentic pride will increase the likelihood of wielding prestige strategies, while hubristic pride will increase the likelihood of wielding dominance strategies (Cheng et al., 2010; Tracy et al., 2010, 2020). Some studies have provided direct evidence for the correlation between pride and social status pursuit strategy: authentic pride is positively correlated with prestige, and hubristic pride is positively correlated with dominance (Cheng et al., 2010; Bolló et al., 2018). In addition, some studies have indirectly supported that authentic pride facilitates the attainment of prestige and hubristic pride facilitates the attainment of dominance. For example, long-distance runners and students with high levels of authentic pride are more likely to change their behaviors to achieve socially valued success (Weidman et al., 2016), which is closer to prestige strategies. However, people with high levels of hubristic pride are more likely to develop prejudices against stigmatized others (Ashton-James and Tracy, 2012) and engage in dishonest behavior (Mercadante and Tracy, 2021), which are closer to dominance strategies.

Furthermore, individuals who adopt different social status strategies have differences in behavior. People who use prestige strategies are more likely to use a specific social influence tactic, relationship building, and less likely to use specific social influence tactics, such as silent treatment, coercion, regression and authority (Ketterman and Maner, 2021). Prestige-oriented individuals may buy their mates an expensive gift to retain them, while dominance-oriented individuals may derogate their mate or behave violently toward sexual rivals (Conlon, 2019). In the field of consumption, previous studies have shown that dominance-oriented male consumers send status signals by consuming large sized products/brands (Panchal and Gill, 2020) and a signal threat to rivals and elicit behavioral avoidance by consuming negative branding (King and Auschaitrakul, 2021).

In the current study, we posit that the social status pursuit strategy used by consumers affects their eWOM behaviors. Positive eWOM can convey positive information to other consumers and help companies increase their reputation among other consumers as well as their profits. Constructive eWOM can better demonstrate the skills and knowledge of consumers because they need to propose problems and solutions that benefit the relevant company. Therefore, prestigious consumers tend to write positive eWOM and constructive eWOM because they typically acquire social status by giving benefits to others (von Rueden et al., 2011). However, dominant consumers tend to write negative eWOM and destructive eWOM because both negative eWOM and destructive eWOM display a threatening image to other consumers and compel a company to pay more to acquire customers by damaging the company's reputation. That is:

H3a: Prestige mediates the relationship between authentic pride and the intention to write positive eWOM.
H3b: Dominance mediates the relationship between hubristic pride and the intention to write negative eWOM.
H4a: Prestige mediates the relationship between authentic pride and the intention to write constructive eWOM.
H4b: Dominance mediates the relationship between hubristic pride and the intention to write destructive eWOM.

To test these predictions, we conducted two studies to test our hypotheses regarding the impact of pride on eWOM behavior. Study 1 manipulated pride states through a recall task and examined the impact of each pride state on different types of eWOM behaviors. Study 2 examined the impact of different types of pride on different types of eWOM behaviors at the trait level and tested whether the social status pursuit strategy mediates these effects.

## Study 1

In Study 1, we manipulated authentic and hubristic pride with a recall task and compared the impact of each pride state on eWOM behavior in a simulated situation. We hypothesized that in a mixed consumption experience (including both positive and negative experiences), participants experiencing authentic pride are more likely to write positive eWOM and constructive eWOM, while participants experiencing hubristic pride are more likely to write negative eWOM and destructive eWOM.

### Method

Two hundred eighty-nine participants from Credamo completed this study. Seventeen participants were excluded because the content they recalled in the recall task did not meet the inclusion requirements (e.g., the participants did not recall his or her own experience, the recalled experience could not be regarded as success, and the participant attributed his or her success to effort in the hubristic pride group). Ultimately, 272 participants remained (67% female; M_age_ = 29).

First, the participants completed the state version of the Authentic and Hubristic Pride Scales as a pretest (Ashton-James and Tracy, 2012). These scales ask participants to rate the extent to which they currently feel each of the 14 affective states as the Trait version of these scales (Tracy and Robins, 2007; e.g., “currently, I feel successful,” 1 = not at all, 5 = very much).

Then, the participants were randomly assigned to one of three conditions (authentic pride vs. hubristic pride vs. control) and completed a recall task [adapted from Ashton-James and Tracy (2012)] that was designed to induce either authentic pride, hubristic pride, or a neutral emotional state. We induced different pride states by directly manipulating participants' attributions for success. Specifically, the participants in the authentic pride condition were asked to recall a time when they were doing truly well in their courses or work because of their efforts (unstable, controllable, and specific attribute). The participants in the hubristic pride condition were asked to recall a time when they were doing truly well in their courses or work because of their talents (stable, uncontrolled, and global attribute). The participants in the control condition were simply asked to recall everything that they had done that day. In addition, the participants completed the state version of the Authentic and Hubristic Pride Scales again as a manipulation check (Ashton-James and Tracy, 2012).

Then, we measured the participants' intention to engage in eWOM behavior based on a simulated hotel stay experience (Yen and Tang, 2019). To motivate the participants to write various eWOM in the scenario, we set up a scenario that contained both positive and negative experiences. Considering sensitivity to negative information, all core attributes were positive, while the facilitating attributes were negative:

*You approached the front desk to check-in. The front desk employee helped you check in and gave you the room card in a few seconds. Then the clerk asked if you needed any assistance and guided you to your room. You thought the staff were very friendly*.

*Walking through the lobby, you found that the lobby and other public areas were beautifully designed and well maintained. Entering your room, you noticed the room door had a security latch. The room smelled fresh, the carpet was vacuumed, and there was no dust on the furniture. Everything in the room was nicely arranged. You felt very relaxed*.

*On the table in your room, you noticed some free snacks: a bottle of water, a piece of chocolate and some baked biscuits. These snacks were well packed and the biscuits tasted delicious. You also found a complimentary relaxation CD on the side table. However, when you tried to play the CD, you found that the CD player made some noise. The next morning when you went to have breakfast, you found that the food you wanted to eat had been taken by others and you had to wait 15 minutes*.

We measured the participants' intention to write four types of eWOM by using a seven-point Likert scale, including “I will write positive comments about this hotel,” “I will write negative comments about this hotel,” “I will write comments about this hotel to help them improve,” and “I will write comments about this hotel to punish them for their bad work” (1 = strongly impossible, 7 = strongly possible).

### Results and Discussion

#### Manipulation Checks

Before the recall task, there was no significant difference among the three groups in both authentic pride and hubristic pride (Authentic pride: *M*_authentic_ = 3.68, *SD* = 0.82; *M*_hubristic_ = 3.76, *SD* = 0.70; *M*_control_ = 3.62, *SD* = 0.80; *F* (2, 269) = 0.730, *p* > 0.05; Hubristic pride: *M*_authentic_ = 2.03, *SD* = 0.73; *M*_hubristic_ = 2.02, *SD* = 0.69; *M*_control_ = 1.86, *SD* = 0.53; *F* (2, 269) = 2.028, *p* > 0.05). After the recall task, the participants in the authentic pride condition reported higher levels of authentic pride (*M* = 4.45, *SD* = 0.35) than the participants in the hubristic pride condition (*M* = 4.27, *SD* = 0.40; *t* (178) = 3.253, *p* < 0.01) and the control condition (*M* = 3.78, *SD* = 0.81; *t* (180) = 7.205, *p* < 0.001). The participants in the hubristic pride condition reported higher levels of hubristic pride (*M* = 2.50, *SD* = 0.89) than the participants in the authentic pride condition (*M* = 1.99, *SD* = 0.82; *t* (178) = 3.949, *p* < 0.001) and the control condition (*M* = 1.79, *SD* = 0.56; *t* (180) = 6.425, *p* < 0.001).

#### Main Effect

A series of multivariate analyses of variance (MANOVA) revealed that pride states have a significant impact on positive eWOM (*F* (2, 267) = 6.904, *p* < 0.01), negative eWOM (*F* (2, 267) = 6.982, *p* < 0.01), constructive eWOM (*F* (2, 267) = 7.594, *p* < 0.01) and destructive eWOM (*F* (2, 267) = 5.607, *p* < 0.01). Specifically, the intention to write positive eWOM was distinctly higher in the authentic pride condition (vs. hubristic pride condition, *p* < 0.001; vs. control condition, *p* < 0.05), but there were no significant differences between the hubristic pride condition and the control condition. The intention to write negative eWOM was distinctly higher in the hubristic pride condition (vs. authentic pride condition, *p* < 0.01; vs. control condition, *p* < 0.01), but there were no significant differences between the authentic pride condition and the control condition. The intention to write constructive eWOM was distinctly higher in the authentic pride condition (vs. hubristic pride condition, *p* < 0.001; vs. control condition, *p* < 0.01), but there were no significant differences between the hubristic pride condition and the control condition. The intention to write destructive eWOM was distinctly higher in the hubristic pride condition (vs. authentic pride condition, *p* < 0.01; vs. control condition, *p* < 0.05), but there were no significant differences between the authentic pride condition and control condition (see Table 1). These results supported H1a, H1b, H2a and H2b.

**Table 1:** Means between the pride conditions: study 1.

	**Authentic pride**	**Hubristic pride**	**Control**	***F***	***p***
Positive eWOM	4.91 (0.94)	4.26 (1.38)	4.48 (1.14)	6.904	0.001ac
Negative eWOM	2.91 (1.02)	3.57 (1.33)	3.02 (1.36)	6.982	0.001bc
Constructive eWOM	6.07 (0.85)	5.53 (1.03)	5.62 (1.09)	7.594	0.001ac
Destructive eWOM	3.01 (1.33)	3.71 (1.49)	3.20 (1.46)	5.607	0.004bc

*a, Authentic pride condition significantly different from control condition, p < 0.05*.

*b, Hubristic pride condition significantly different from control condition, p < 0.05*.

*c, Authentic pride condition significantly different from hubristic pride condition, p < 0.05*.

## Study 2

Study 2 explored the impact of the two facets of pride on different eWOM behaviors at the trait level and tested the mediating role of social status pursuit strategy in this process. We hypothesized that participants with high levels of authentic pride would be more inclined to use a prestige strategy and would thus be more likely to write positive eWOM and constructive eWOM, while participants with high levels of hubristic pride would be more inclined to use a dominance strategy and would therefore be more likely to write negative eWOM and destructive eWOM.

### Method

Four hundred and ten participants from Credamo completed this study. Thirteen participants were excluded because they failed the attention check. Ultimately, 397 participants remained (49% female; M_age_ = 28).

First, we measured pride, the social status pursuit strategy and eWOM behaviors with measures derived from previous studies. Authentic pride and hubristic pride were measured on the trait version of the Authentic and Hubristic Pride Scales developed by Tracy and Robins (2007). The scale contains two dimensions and a total of 14 items. Seven items were used to measure authentic pride (α = 0.92), and the other seven items were used to measure hubristic pride (α = 0.88). Prestige and dominance were measured on the 17-item scale revised by Cheng et al. (2010). The scale contains two dimensions and a total of 17 items. Nine items were used to measure prestige; however, based on a reliability analysis and confirmatory factor analysis (CFA), we excluded three items and retained only six items (α = 0.86) for the current study. The other eight items in the scale were used to measure dominance; however, based on a reliability analysis and CFA, we excluded two items and retained only six items (α = 0.87) for the current study. Participants' intentions to write positive eWOM or negative eWOM were measured on a 6-item scale adapted by Fu et al. (2015). In this scale, three items were used to measure the intention to write positive eWOM (α = 0.89), and the other three items were used to measure the intention to write negative eWOM (α = 0.90). Following Weitzl (2019), this study used typical and specific constructive WOM behavior (helping a company) and destructive WOM behavior (revenge taking) to reflect the participants' intentions to write constructive or destructive eWOM. Helping a company was measured on a 4-item scale adapted from Hennig-Thurau et al. (2004). Based on a reliability analysis and CFA, we excluded one item and retained only three items (α = 0.76) for the current study. Revenge taking was measured on a 3-item scale developed by Wetzer et al. (2007; α = 0.90).

In addition, to better reflect the impacts of the two strategies on consumers' constructive and destructive eWOM, and to reduce common method bias, we conducted measurements at the behavioral level. To make participants equally likely to write constructive eWOM and destructive eWOM, participants were asked to recall an unsatisfactory consumption experience and then to write an online review based on this negative experience. Two graduate students who were unaware of the hypotheses coded the reviews written by the participants; the answers were coded as constructive eWOM, destructive eWOM, neither, or as error responses. Answers that merely described an experience and did not reflect constructiveness or destructiveness were coded as neither. Some participants did not write a review as required or recalled a positive consumption experience and then commented on it. These answers were coded as error responses. The Cohen's kappa value between the two independent coders was 0.82, and a third coder, unaware of the hypotheses, resolved the discrepancies.

### Results and Discussion

#### Preliminary Analyses

Table 2 provided the means and standard deviations for all measures employed alongside the correlations among all constructs.

**Table 2:** Descriptive statistics, correlations and Cronbach's α.

**Variables**	**GDR**	**AGE**	**AP**	**HP**	**P**	**D**	**PE**	**NE**	**HC**	**RT**
GDR	**N/A**									
AGE	0.06	**N/A**								
AP	−0.23***	0.28***	**0.92**							
HP	−0.01	−0.04	−0.04	**0.88**						
P	−0.23***	0.23***	0.74***	0.04	**0.86**					
D	−0.07	−0.05	−0.06	0.66***	0.09	**0.87**				
PE	−0.07	0.19***	0.54***	−0.03	0.56***	0.03	**0.89**			
NE	0.07	−0.13**	−0.10	0.34***	−0.12*	0.33***	0.06	**0.90**		
HC	−0.09	0.11*	0.39***	−0.06	0.42***	−0.03	0.46***	−0.05	**0.76**	
RT	0.04	−0.25***	−0.38***	0.34***	−0.33***	0.33***	−0.32***	0.31***	−0.43**	**0.90**
*M*	1.49	28.37	5.30	2.82	5.08	3.03	5.36	3.51	5.25	2.71
*SD*	0.50	5.63	0.97	1.12	0.89	1.16	1.20	1.66	1.12	1.41

***p < 0.001, **p < 0.01, *p < 0.05, Bold face items on the diagonal are the Cronbach's α GDR, gender, AP, authentic pride; HP, hubristic pride; P, prestige; D, dominance; PE, positive eWOM; NE, negative eWOM; HC, helping a company; RT, revenge taking.

#### Main Effect

Hypotheses 1a, 1b, 2a and 2b were tested using multiple linear regressions. Regression analysis revealed that after controlling for gender and age, authentic pride positively affected participants' intention to engage in positive eWOM (β = 0.55, *p* < 0.001) and their intention to help the company (β = 0.39, *p* < 0.001), thereby supporting H1a and H2a. Hubristic pride positively affected participants' intention to engage in negative eWOM (β = 0.34, *p* < 0.001) and their intention to take revenge (β = 0.33, *p* < 0.001), thereby supporting H1b and H2b.

#### Mediation Effects

Hypotheses 3a, 3b, 4a and 4b were tested using Baron and Kenny's (1986) method. Regarding H3a and H4a, a previous analysis showed that authentic pride significantly predicted the intentions to write positive eWOM and help a company. Further analysis showed that authentic pride significantly predicted prestige (β = 0.71, *p* < 0.001) and that prestige significantly predicted the intentions to write positive eWOM (β = 0.56, *p* < 0.001) and help a company (β = 0.42, *p* < 0.001). Multiple regressions showed that both authentic pride (β = 0.29, *p* < 0.001) and prestige (β = 0.36, *p* < 0.001) significantly predicted writing positive eWOM, thereby supporting H3a. Both authentic pride (β = 0.18, *p* < 0.05) and prestige (β = 0.30, *p* < 0.001) significantly predicted helping a company, thereby supporting H4a.

For H3b and H4b, a previous analysis showed that hubristic pride significantly predicted the intentions to write negative eWOM and take revenge. Further analysis showed that hubristic pride significantly predicted dominance (β = 0.66, *p* < 0.001) and that dominance significantly predicted the intentions to write negative eWOM (β = 0.33, *p* < 0.001) and take revenge (β = 0.32, *p* < 0.001). Multiple regressions showed that both hubristic pride (β = 0.21, *p* < 0.01) and dominance (β = 0.19, *p* < 0.01) significantly predicted writing negative eWOM, thereby supporting H3b. Both hubristic pride (β = 0.21, *p* < 0.01) and dominance (β = 0.19, *p* < 0.01) significantly predicted revenge taking, thereby supporting H4b.

#### Behavioral Analysis

Forty-seven answers that were coded as error responses were removed, and 350 responses were left to be analyzed. First, we further explored the impact of authentic pride on the intention to write constructive eWOM. We conducted a binomial logistic regression with perceived authentic pride as the independent variable, constructive eWOM (Yes = 1, No = 0) as the dependent variable, and gender and age as the control variables. The results showed that authentic pride significantly predicted constructive eWOM (*B* = 0.46, *p* < 0.001). Next, we further tested the mediating role of prestige and found that authentic pride significantly predicted prestige (*B* = 0.63, *p* < 0.001) and that prestige significantly predicted constructive eWOM (*B* = 0.55, *p* < 0.001). A binomial logistic regression with authentic pride and prestige as the independent variables showed that prestige significantly predicted constructive eWOM (*B*= 0.40, *p* < 0.05), while authentic pride had no significant effect on constructive eWOM (*B* = 0.21, *p* > 0.05). The results thus supported H2a and H4a, which is in line with the results of the self-reported scales.

Second, we further explored the impact of hubristic pride on the intention to write destructive eWOM. We conducted a binomial logistic regression with perceived hubristic pride as the independent variable, destructive eWOM (Yes = 1, No = 0) as the dependent variable, and gender and age as the control variables. The results showed that hubristic pride significantly predicted destructive eWOM (*B* = 0.36, *p* < 0.001). Next, we further tested the mediating role of dominance and found that hubristic pride significantly predicted dominance (*B* = 0.67, *p* < 0.001) and that dominance significantly predicted destructive eWOM (*B* = 0.37, *p* < 0.001). A binomial logistic regression with hubristic pride and dominance as the independent variables showed that the correlation between dominance and destructive eWOM was marginally significant (*B* = 0.24, *p* = 0.064), while hubristic pride had no significant effect on destructive eWOM (*B* = 0.20, *p* > 0.05). The results therefore partly support H2b and H4b, which is in line with the results of the self-reported scales.

## Discussion

The development of online review platforms and social networking sites provides not only assistance to consumers in product evaluation and purchase decisions by generating and spreading eWOM but also a new space for consumers to build their self-image and establish interpersonal relationships. Consumers' eWOM on social networks extends beyond their interactions with products and their interactions with service personnel. Expectations of how eWOM will affect other consumers' perceptions of them also impact consumers' eWOM behaviors. Therefore, it is important to explore consumers' eWOM behaviors in the context of social interactions. This study thus explored how two different subtypes of pride influence consumers' eWOM through different social status pursuit strategies. Our results show that authentic pride promotes positive and constructive eWOM through the mediator of prestige, while hubristic pride promotes negative and destructive eWOM through the mediator of dominance.

### Theoretical Implications

The current research extends the previous literature in several ways. First, this research adds to our understanding of different types of eWOM. Consumers have different willingness to write different types of eWOM. The results of both study 1 and study 2 show that consumers are more willing to write positive eWOM and constructive eWOM and more reluctant to write negative eWOM and destructive eWOM. Furthermore, we find that one factor may have different impacts on consumers' willingness to post different types of eWOM. Past research has largely focused on the drivers of overall eWOM or a specific type of eWOM (Hu and Kim, 2018; Nam et al., 2020; Sohaib et al., 2020; Kim and Hwang, 2022). In the current research, we explore how emotions influence consumers' willingness to write various types of eWOM differently. We also focus on a pair of eWOM, constructive and destructive eWOM (Wetzer et al., 2007). Limited attention has been given to constructive and destructive eWOM, and there is a lack of empirical research on its antecedent variables (Weitzl, 2019). However, as consumers gain increasing influence in the market, constructive eWOM, as a typical behavior of value-cocreatin, and destructive eWOM, as a typical behavior of value- codestruction, are more important. This research empirically demonstrates that when consumers feel authentic pride, they prefer to write constructive eWOM, while consumers feel hubristic pride, they prefer to write destructive eWOM. In addition, we provide a novel method to measure the willingness to write constructive eWOM or destructive eWOM by asking participants to write a review on an unsatisfactory consumer experience.

Second, this research furthers our understanding of how consumers' emotions, specifically, their discrete positive emotions, affect their eWOM behaviors. Our results indicate that authentic pride and hubristic pride encourage consumers to create different types of eWOM to express their consumption experiences, thereby reflecting how discrete positive emotions can have a unique impact on individuals' cognition, expression, and behavior (Griskevicius et al., 2010; Campos et al., 2013; Shiota et al., 2014; Nikolinakou and King, 2018a). Furthermore, our research enriches previous studies by showing that differences within similar discrete positive emotions can impact the expressions and behaviors of consumers. Our research also shows that evolution-based mechanisms still function in the emerging field of eWOM. From an evolutionary perspective, previous studies have regarded discrete emotions as neurocomputational programs that solve adaptive problems by activating a series of cognitive and motivational subprograms (Griskevicius et al., 2010; Sznycer et al., 2017). Our results indicate that pride influences consumers' intentions to write different types of eWOM through their pursuit of social status, which is an adaptive behavior. Thus, once again, this finding validates those of previous studies. In addition, we provide empirical support for the manipulation of pride (Ashton-James and Tracy, 2012). Our results show that the manipulation of pride can indeed increase participants' authentic pride and hubristic pride by measuring their pride before and after manipulation.

Finally, this research contributes to the dominance-prestige model (Henrich and Gil-White, 2001; Cheng et al., 2013). This model suggests that the pursuit of social status includes dominance strategies, which are common in primates, and prestige strategies, which are unique to humans. Previous studies have explored how dominance strategies and prestige strategies are linked to different personality variables (Cheng et al., 2010), mate retention strategies (Conlon, 2019), specific social influence tactics (Ketterman and Maner, 2021) and consumer behaviors (Panchal and Gill, 2020; King and Auschaitrakul, 2021). Our research expands this investigation to the context of eWOM behaviors and finds that prestige strategies promote positive eWOM and constructive eWOM, while dominance strategies cause negative eWOM and destructive eWOM. Our results also provide support for the temporal stability of the dominance-prestige model. eWOM is a field that has only emerged in recent decades, and the dominance-prestige model still explains people's behaviors in this emerging field. This result implies that the model remains stable even as the environment changes.

### Practical Implications

The volume of eWOM can positively affect perceived credibility and a company's revenue (Filieri, 2015; Yan et al., 2018; Kim et al., 2019). However, different types of eWOM have different impacts on consumers (Lis and Fischer, 2020). For example, positive eWOM and constructive eWOM, as typical behaviors of value-cocreatin, can bring benefits to businesses, while negative eWOM and destructive eWOM, as typical behaviors of value-codestruction, will harm the merchants (Dolan et al., 2019). Study 1 showed that consumers may write different types of eWOM for the same consumer experience. Therefore, when firm and marketers are engaging in social media marketing, they need to be aware of which type of eWOM consumers are more likely to generate and then induce the specific types of eWOM, such as positive eWOM and constructive eWOM.

Previous research has shown that pride has a significant impact on eWOM creation intention and has suggested that firms provide services that trigger the emotion of pride (Wen et al., 2018). However, our results show that only triggering authentic pride can generate more eWOM that is good for the company, and triggering hubristic pride will generate more negative eWOM and destructive eWOM, which is detrimental to the company. Therefore, marketers should be careful to stimulate consumer pride. They need to further consider which marketing strategies activate the authentic pride of consumers and which marketing strategies activate the hubristic pride of consumers.

Our results also show that status strategy is an important antecedent variable related to how consumers write different types of eWOM. Individuals who tend to use a prestige strategy are more likely to engage in value-cocreation, i.e., positive and constructive eWOM, while consumers who tend to use a dominance strategy are more likely to engage in value-codestruction represented by negative and destructive eWOM. The results therefore suggest that the intention to write eWOM can be effectively affected by influencing consumers' social status pursuit strategies. Finally, based on the theory of planned behavior, we believe that creating a community norm and atmosphere that foster a prestige-based pursuit of social status can better promote positive and constructive eWOM.

### Limitations and Future Directions

This research focuses on the influence of different subtypes of pride on different types of eWOM through social status pursuit strategies. To further understand how discrete positive emotions affect eWOM through adaptive mechanisms, future research should explore the impacts of other discrete positive emotions, such as enjoyment and happiness. In addition, we should also determine whether discrete negative emotions can affect different types of eWOM through social status pursuit strategies. For example, anger is one of the main reasons for aggressive behaviors, which can also lead to revenge taking. Does dominance mediate this process? Future research can identify whether other identifiable variables affect consumers' social status pursuit strategies, their eWOM behaviors, and other consumer behaviors.

Future research can explore the antecedents that influence consumer' intentions to write different types of eWOM more systematically based on the theory of planned behavior (Ajzen, 1991). The current research only examines the mechanism of social status-acquisition strategies, which reflect one of the three determinants of intention, attitude. Individuals who adopted the prestige strategies have more positive attitudes toward positive eWOM and constructive eWOM, while individuals who adopted the dominance strategies have more positive attitudes toward negative eWOM and destructive eWOM. However, subjective norm and perceived behavioral control also influence consumers' intention to write different types of eWOM. For example, consumers are less likely to write negative eWOM when there is a general belief that they should be sympathetic and tolerant of service personnel and when the process for posting a negative eWOM is more complicated. Future research can further explore the antecedents of posting different types of eWOM from the perspective of subjective norm and perceived behavioral control.

In addition, although the current research discusses the influence of pride on eWOM from the perspectives of state pride and trait pride, both the manipulation and questionnaire measurement are different from those used in real situations. Future researchers can influence consumer pride through product design and analyze the content posted by users online to identify their pride and social status pursuit strategies to more realistically predict consumers' eWOM behaviors.

Finally, in this research, the participants were asked to write eWOM in a simulated situation or a recalled situation, which is different from an actual scenario. Therefore, there may be differences between the participants' consumer experiences and actual consumer experiences, especially in terms of emotional arousal. Future research can therefore use field studies to manipulate participants' pride directly, either before or during consumption, or to measure their emotions immediately after they create comments to enhance the external validity.

## Data Availability Statement

The raw data supporting the conclusions of this article will be made available by the authors, without undue reservation.

## Ethics Statement

The studies involving human participants were reviewed and approved by Zhejiang University. The patients/participants provided their written informed consent to participate in this study.

## Author Contributions

JL designed the study and drafted the initial manuscript. QX collected the data and performed statistical analysis. JZ contributed to the revised manuscript. All authors discussed the results and contributed to the final manuscript.

## Conflict of Interest

The authors declare that the research was conducted in the absence of any commercial or financial relationships that could be construed as a potential conflict of interest.

## Publisher's Note

All claims expressed in this article are solely those of the authors and do not necessarily represent those of their affiliated organizations, or those of the publisher, the editors and the reviewers. Any product that may be evaluated in this article, or claim that may be made by its manufacturer, is not guaranteed or endorsed by the publisher.
